# Natural Killer Cell Activation Signature Identifies Cyclin B1/CDK1 as a Druggable Target to Overcome Natural Killer Cell Dysfunction and Tumor Invasiveness in Melanoma

**DOI:** 10.3390/ph18050666

**Published:** 2025-04-30

**Authors:** Linbin Chen, Wanqian Liao, Jing Huang, Qiuyue Ding, Junwan Wu, Qiong Zhang, Ya Ding, Dandan Li, Jingjing Li, Xizhi Wen, Xiaoshi Zhang

**Affiliations:** 1 State Key Laboratory of Oncology in South China, Collaborative Innovation Center for Cancer Medicine, Sun Yat-sen University Cancer Center, Guangzhou 510060, China; chenlb@sysucc.org.cn (L.C.); liaowq@sysucc.org.cn (W.L.); huangjing1@sysucc.org.cn (J.H.); dingqy2@sysucc.org.cn (Q.D.); wujw2@sysucc.org.cn (J.W.); zhangqiong@sysucc.org.cn (Q.Z.); dingya@sysucc.org.cn (Y.D.); lidd@sysucc.org.cn (D.L.); lijingj@sysucc.org.cn (J.L.); 2 Department of Biological Therapy Center, Sun Yat-sen University Cancer Center, Guangzhou 510060, China; 3 Department of Medical Oncology, Sun Yat-sen University Cancer Center, Guangzhou 510060, China

**Keywords:** melanoma, NK cells, CDK inhibitors, STAT3 signaling pathway, PD-L1

## Abstract

**Background/Objectives:** Natural killer (NK) cells play a crucial role in immune surveillance against melanoma, yet they frequently exhibit dysfunction in the tumor microenvironment. This study aims to establish an NK cell activation-related prognostic signature and identify potential druggable targets to overcome NK cell dysfunction. **Methods**: A prognostic signature was developed using the TCGA-SKCM cohort and validated across independent datasets. NK cell activation and cytotoxicity were evaluated in melanoma-NK-92MI co-culture systems via flow cytometry. Mechanistic studies employed Western blotting, co-immunoprecipitation, ELISA, and qRT-PCR. Single-cell RNA-seq data were used to analyze cell–cell communication. **Results**: A four-gene NK cell activation signature was identified and validated for prognostic significance across five independent melanoma datasets. Among the identified genes, cyclin B1 (CCNB1) emerged as a novel therapeutic target for overcoming NK cell resistance. In vivo, pharmacological inhibition of the CCNB1/Cyclin-dependent kinase 1 (CDK1) complex with RO-3306 significantly suppressed melanoma growth by enhancing NK cell infiltration and IFN-γ production. In vitro, CCNB1 knockdown in melanoma cells augmented NK-92MI activation, as evidenced by increased expression of CD69, CD107a, IFN-γ, and NKG2D, thereby improving NK cell-mediated cytotoxicity. Mechanistically, in melanoma cells, the CCNB1/CDK1 complex phosphorylates STAT3, activating the IL-6/STAT3 positive feedback loop, which upregulates PD-L1 and enables resistance to NK cell-mediated cytotoxicity. Beyond its role in immune evasion, CCNB1 also promoted melanoma invasiveness by inducing epithelial–mesenchymal transition (EMT) through the TGF-β-SMAD2/3 signaling. **Conclusions**: This study establishes CCNB1/CDK1 as a novel immunotherapeutic target and uncovers a new role for CDK1 inhibitors in enhancing NK cell function and suppressing melanoma progression.

## 1. Introduction

Melanoma is a malignancy arising from the malignant transformation of skin melanocytes and represents the most lethal type of skin cancer [1,2]. Although melanoma accounts for a smaller proportion of all skin cancers, its aggressive nature and rapid metastasis make it one of the leading causes of cancer-related deaths worldwide [3]. In recent years, the clinical application of immune checkpoint inhibitors, such as anti-PD-1/CTLA-4 antibodies, has led to significant advancements in melanoma treatment [4,5]. However, many patients either develop resistance to immune therapy or fail to respond altogether, highlighting the urgent need to explore new therapeutic strategies. Studies have shown that tumor immune evasion mechanisms contribute significantly to the difficulty of treating melanoma [6,7]. Therefore, enhancing immune cell activity, particularly that of T cells and NK cells within the tumor microenvironment (TME), has become a critical focus of current research.

NK cells, as an essential component of the innate immune system, play a vital role in anti-tumor immunity by directly recognizing and killing tumor cells. Unlike T cells, NK cells do not rely on antigen-presenting cells for activation. Instead, they recognize tumor cells through the absence of major histocompatibility complex I (MHC-I) molecules on the surface, release cytokines such as IFN-γ, and utilize receptor-mediated cytotoxicity to directly destroy tumor cells [8]. However, immune suppressive factors within the TME, such as activation of immune checkpoints and secretion of cytokines, often inhibit NK cell function, leading to tumor immune evasion [9,10].

Currently, numerous studies focus on engineering NK cells to enhance their therapeutic efficacy [11,12]. However, tumor cells have intricate intrinsic mechanisms to resist NK cell-mediated cytotoxicity [13]. Therefore, there is growing interest in strategies to make cancer cells more vulnerable to NK cell-mediated killing. In this study, we constructed a risk model based on NK cell activation-related genes and identified CCNB1 as a druggable target driving melanoma resistance to NK cell-mediated cytotoxicity. In vivo, pharmacological inhibition of CCNB1 enhanced NK cell infiltration and activation. In vitro mechanistic studies demonstrated that the CCNB1/CDK1 complex activates the STAT3/PD-L1 pathway, leading to NK cell dysfunction. Meanwhile, CCNB1 upregulates TGF-β expression and secretion, inducing EMT and enhancing melanoma invasiveness. These findings highlight CCNB1/CDK1 as a key regulator of NK cell-mediated immune evasion and a promising target for melanoma immunotherapy.

## 2. Results

### 2.1. Construction of Prognostic Signature Associated with NK Cell Activation

**Figure 1:**
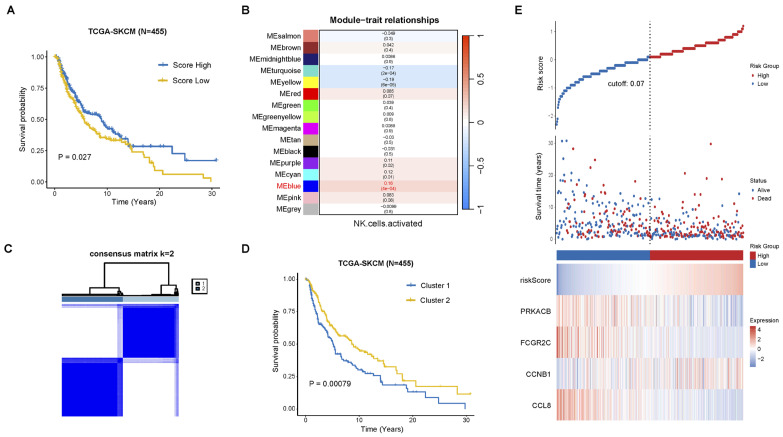
Prognostic model of NK cell activation-related genes. (**A**) Kaplan–Meier analysis in the TCGA-SKCM cohort shows the correlation between the infiltration level of activated NK cells and overall survival (OS). Patients are divided into high and low infiltration groups based on the median infiltration level of activated NK cells calculated by CIBERSORT. (**B**) WGCNA identifies NK cell activation-related modules. (**C**) Consensus clustering identifies two optimal clusters. (**D**) Kaplan–Meier analysis shows significant overall survival differences between the two clusters. (**E**) The “ggrisk” R package version 1.3 (cutoff = 0.07) is used to classify high- and low-risk groups in the TCGA-SKCM cohort.

We analyzed immune cell infiltration in TCGA-SKCM patients (*n* = 455) using CIBERSORT and stratified them into high and low infiltration groups based on median scores. Patients with higher NK cell activation scores had significantly better overall survival (OS) (*p* = 0.027, Figure 1A). Using Weighted gene co-expression network analysis (WGCNA), we identified gene modules associated with NK cell activation, selecting a soft threshold of 6 for network construction (Figure S1A,B). The MEblue module showed the strongest positive correlation (cor = 0.16, *p* = 0.0004, Figure 1B). Univariate Cox regression identified OS-related genes from the MEblue module, which were used for consensus clustering, revealing two distinct clusters (Figure 1C and Figure S1C–E). Cluster 1 had significantly poorer OS than Cluster 2 in TCGA-SKCM (*p* = 0.00079, Figure 1D).

Furthermore, we identified differentially expressed genes between the two clusters through univariate Cox regression analysis. By overlapping these genes with the “innate immunity” gene set from GeneCards, we obtained 457 NK activation-related genes (NKAGs). These NKAGs were subjected to a Least absolute shrinkage and selection operator (LASSO) regression model for feature selection, followed by stepwise regression to pinpoint four key prognostic genes associated with NK cell activation (Figure S1F,G). Among these, PRKACB, FCGR2C, and CCL8 were identified as protective genes, whereas CCNB1 was classified as a risk gene (Figure 1E). The risk score for each patient was calculated using the following formula: Risk score = CCNB1 × 0.35865 − PRKACB × 0.22772 − FCGR2C × 0.21722 − CCL8 × 0.41216.

### 2.2. Functional and Immune Infiltration Analysis of the Prognostic Model

**Figure 2:**
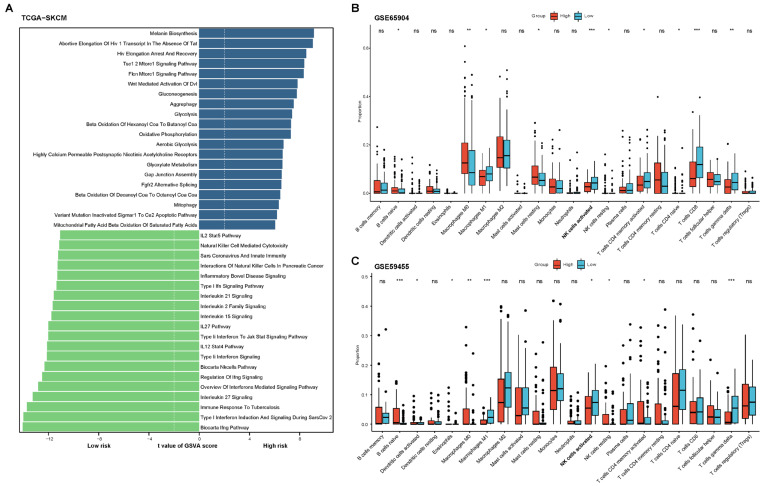
Functional and immune infiltration analysis of the prognostic model. (**A**) GSVA analysis was performed on the TCGA-SKCM cohort to identify the top 20 enriched MSigDB_C2 pathways in the high-risk and low-risk groups. (**B**,**C**) Immune cell infiltration levels between the high-risk and low-risk groups were compared in melanoma cohorts GSE65904 and GSE59455 using the CIBERSORT algorithm. ns, not significant; * *p* < 0.05; ** *p* < 0.01, *** *p* < 0.001.

We conducted GSVA analysis on the TCGA-SKCM dataset to identify activated pathways in the high-risk and low-risk groups. Figure 2A highlighted the top 20 enriched pathways for each group. In the low-risk group, several key pathways related to NK cell activation were enriched, including type I and type II interferon signaling, IL-12/STAT4 and IL-15 signaling, and Natural Killer Cell-mediated cytotoxicity. These pathways were crucial for regulating NK cell activation, proliferation, and cytotoxic activity in response to immune challenges such as viral infections and cancer. Additionally, IL-27 and IL-2 family signaling also played important roles in modulating NK cell responses.

CIBERSORT analysis of GSE65904 and GSE59455 revealed higher NK cell activation scores and fewer resting NK cells in the low-risk group (Figure 2B,C). Furthermore, the infiltration of M1 polarized macrophages was also increased in the low-risk group. In the GSE65904 cohort, the proportion of CD8^+^ T cell infiltration was also elevated in the low-risk group. These findings suggested that low-risk patients exhibit stronger anti-tumor immune cell infiltration.

### 2.3. Validation of the Prognostic Model in Multiple Cohorts

**Figure 3:**
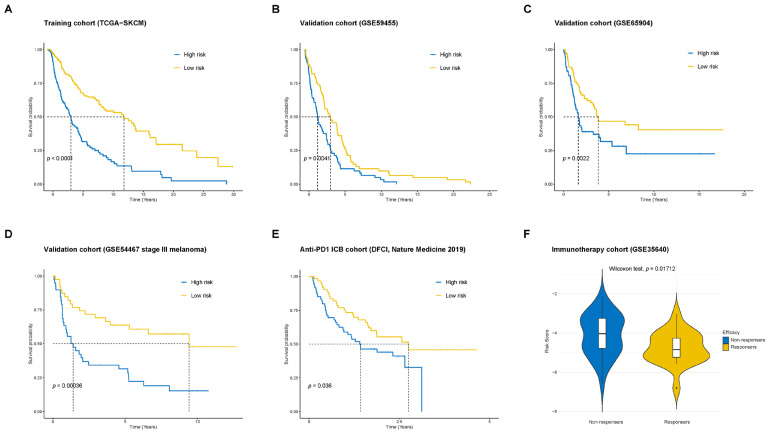
Validation of the prognostic model. (**A**) Validation of OS differences between the high-risk and low-risk groups in the training cohort, TCGA-SKCM. (**B**,**C**) Validation of OS differences between the high-risk and low-risk groups in the melanoma cohorts GSE59455 and GSE65904. (**D**) Validation of OS differences between the high-risk and low-risk groups in the stage III melanoma cohort, GSE54467. (**E**) Validation of OS differences between the high-risk and low-risk groups in the DFCI cohort of patients receiving anti-PD-1 therapy. (**F**) Comparison of risk scores between responders and non-responders in the MAGE-A3 immunotherapy cohort, GSE35640.

To validate the prognostic model, we utilized several independent cohorts, including the training cohort TCGA-SKCM, conventional melanoma cohorts (GSE59455 and GSE65904), a stage III melanoma cohort (GSE54467), the anti-PD1 immune checkpoint blockade (ICB) cohort, and the MAGE-A3 immunotherapy cohort. Our analysis demonstrated that the high-risk group consistently exhibited significantly poorer OS and lower immune response compared to the low-risk group, with a statistically significant difference (*p* < 0.05) in each of the cohorts (Figure 3A–F). The receiver operating characteristic (ROC) curves for each cohort, as shown in Figure S2, further support the model’s reliability and potential clinical applicability.

### 2.4. Pharmacological CCNB1 Inhibition (RO-3306) Suppresses Melanoma Growth by Enhancing NK Infiltration and Cytotoxicity In Vivo

**Figure 4:**
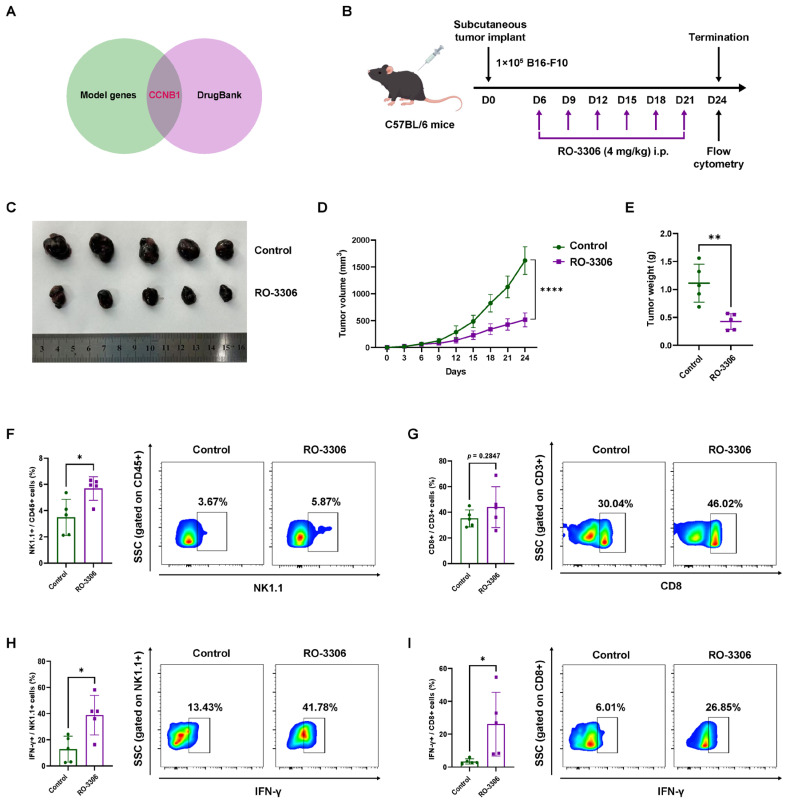
Pharmacological CCNB1 inhibition (RO-3306) suppresses melanoma growth by enhancing NK infiltration and cytotoxicity in vivo. (**A**) Venn diagram showing the identification of CCNB1 as a druggable target by overlapping model genes (PRKACB, FCGR2C, CCL8 and CCNB1) with the DrugBank database. (**B**) Schematic representation of the in vivo experimental design. C57BL/6 mice were subcutaneously implanted with 1 × 10^5^ B16-F10 melanoma cells. RO-3306 (4 mg/kg) was administered intraperitoneally every three days. By Figdraw. (**C**) Representative images of tumors excised from control and RO-3306-treated mice at the experimental endpoint. (**D**,**E**) Tumor volume growth curves and tumor weight measurements. (**F**,**G**) Flow cytometry analysis of the percentage of NK cells (NK1.1^+^CD45^+^) and CD8^+^ T cells (CD3^+^ CD8^+^) in tumors. (**H**,**I**) Percentage of IFN-γ production from NK cells and CD8^+^ T cells in tumors. All data are presented as mean ± s.d. Two-sided Student’s *t*-test was used for statistical analysis. * *p* < 0.05; ** *p* < 0.01, **** *p* < 0.0001; *n* = 5 per group. i.p. intraperitoneal.

We identified CCNB1 as a druggable target for further investigation by intersecting the model-derived genes with druggable target genes listed in DrugBank (https://go.drugbank.com/, accessed on 5 January 2025) (Figure 4A). RO-3306, a selective small-molecule inhibitor of CCNB1/CDK1, has been widely used in cancer-related research [14,15]. To evaluate its antitumor effects in melanoma, we administered RO-3306 to C57BL/6 mice bearing subcutaneous B16-F10 melanoma (Figure 4B) and observed a significant suppression of tumor growth (Figure 4C–E). Flow cytometry analysis revealed a marked increase in intratumoral NK cell infiltration in the RO-3306-treated group (Figure 4F), while CD8^+^ T cell infiltration showed no significant difference (Figure 4G). Additionally, NK cells and CD8^+^ T cells in the RO-3306-treated tumors exhibited significantly elevated IFN-γ production (Figure 4H,I). These findings demonstrated that RO-3306 suppresses melanoma growth by enhancing the antitumor immune microenvironment.

### 2.5. CCNB1 Knockdown in Melanoma Cells Enhances NK-92MI Activation and Cytotoxicity

**Figure 5:**
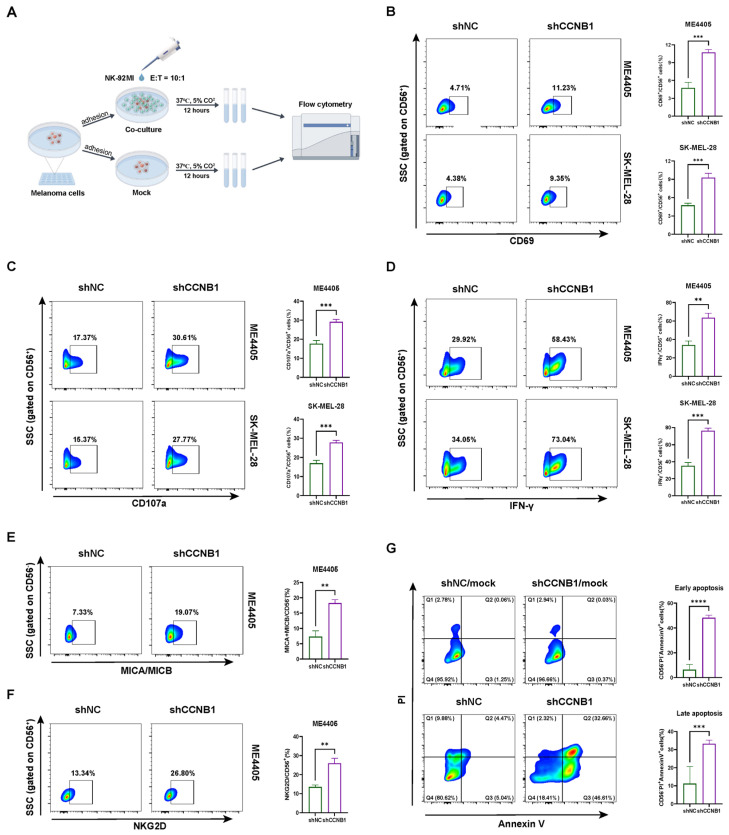
CCNB1 knockdown in melanoma cells enhances NK-92MI activation and cytotoxicity. (**A**) Schematic diagram of co-culturing melanoma cells with NK-92MI cells. By Figdraw. (**B**–**D**) Flow cytometry analysis of CD56^+^ cells in ME4405 or SK-MEL-28 melanoma cells co-cultured with NK-92MI, detecting the levels of CD69, CD107a, and IFN-γ. (**E**) Flow cytometry analysis of CD56^−^ cells in ME4405 co-cultured with NK-92MI, detecting the levels of MICA/MICB. (**F**) Flow cytometry analysis of CD56^+^ cells in ME4405 co-cultured with NK-92MI, detecting NKG2D levels. (**G**) Flow cytometry analysis of CD56^−^ cells in ME4405 co-cultured with NK-92MI, detecting Annexin V/PI levels. The “mock” condition refers to a culture system consisting of ME4405 cells without the addition of NK-92MI cells. All data are presented as mean ± s.d. Two-sided Student’s *t*-test was used for statistical analysis. ** *p* < 0.01; *** *p* < 0.001, **** *p* < 0.0001; *n* = 3 per group.

To further validate the specific role of CCNB1 in mediating melanoma cell resistance to NK cell cytotoxicity, we co-cultured NK-92MI cells with melanoma cells at an E:T ratio of 10:1, as shown in Figure 5A. Flow cytometry was used to assess changes in NK activation markers and cytotoxic effector. In co-culture with two melanoma cell lines, ME4405 and SK-MEL-28 (which harbor BRAF and NRAS mutations, respectively), we observed that knockdown of CCNB1 in melanoma cells increased the proportion of NK-92MI expressing CD69 and CD107a (Figure 5B,C), which are key markers of NK cell activation. More directly, the release of IFN-γ from NK-92MI was significantly upregulated in the co-culture with CCNB1 knockdown melanoma cells (Figure 5D). Additionally, MICA/MICB and NKG2D are a known receptor–ligand pair expressed in tumor cells and NK cells, respectively. Our results showed that CCNB1 knockdown led to an upregulation of MICA/MICB expression in melanoma cells, and a corresponding increase in NKG2D expression in NK-92MI cells (Figure 5E,F). To determine whether the changes in these NK-92MI activation markers led to differential killing ability, we performed apoptosis assays on melanoma cells in the co-culture system. As shown in Figure 5G, CCNB1 knockdown in ME4405 cells co-cultured with NK-92MI significantly increased both early and late apoptosis. Importantly, this effect was not directly caused by CCNB1 knockdown, as no significant changes in apoptosis were observed in the mock group without NK-92MI. Together, these results demonstrated that CCNB1 knockdown enhances NK cell activation and cytotoxicity, promoting melanoma cell apoptosis in vitro.

### 2.6. CCNB1/CDK1 Upregulates STAT3-PD-L1 in Melanoma Cells and Secretes IL-6 to Enhance STAT3 Signaling in NK-92 Cells

**Figure 6:**
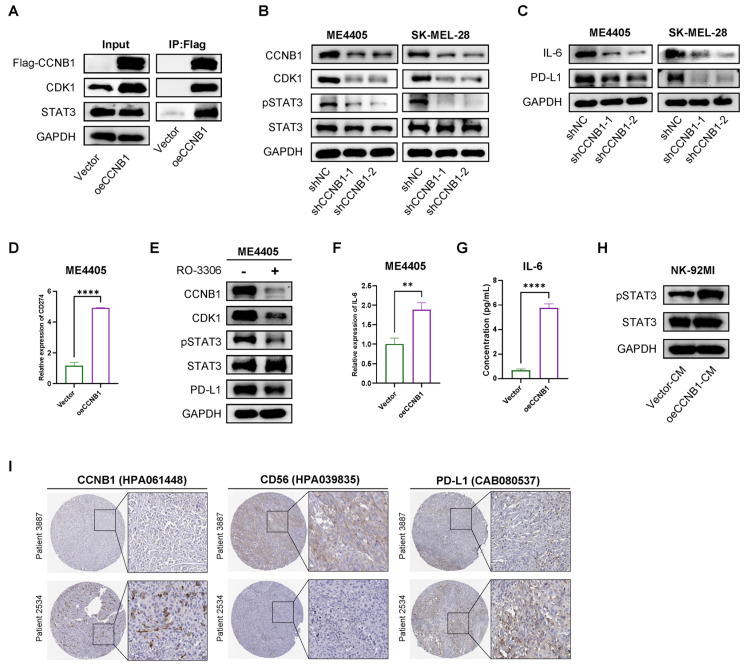
CCNB1/CDK1 upregulates STAT3-PD-L1 in melanoma cells and secretes IL-6 to enhance STAT3 signaling in NK-92 cells. (**A**) Co-immunoprecipitation (Co-IP) analysis of the interaction between CCNB1, CDK1, and STAT3 in ME4405. (**B**) Western blot showing knockdown of CCNB1 decreases CDK1 expression and STAT3 phosphorylation levels in ME4405 and SK-MEL-28 cells. (**C**) Western blot analysis of IL-6 and PD-L1 in ME4405 and SK-MEL-28 following CCNB1 knockdown. (**D**) CD274 mRNA levels in ME4405 cells overexpressing CCNB1 were measured using RT-qPCR. (**E**) Western blot analysis of ME4405 cells treated without or with RO-3306 5 uM for 24 h. (**F**) IL-6 mRNA levels in ME4405 cells overexpressing CCNB1 were measured using RT-qPCR. (**G**) ELISA analysis of IL-6 levels in the supernatant of ME4405 cells overexpressing CCNB1. (**H**) NK-92MI cells were treated for 12 h with conditioned medium from CCNB1-overexpressing ME4405 cells, followed by WB analysis of the STAT3 pathway. (**I**) Representative immunohistochemistry (IHC) staining images of CCNB1, CD56, and PD-L1 expression in melanoma tissues obtained from the Human Protein Atlas (HPA) database. All data are presented as mean ± s.d. Two-sided Student’s *t*-test was used for statistical analysis. ** *p* < 0.01; **** *p* < 0.0001; Fold changes were based on three replicates. CM, conditioned medium.

CCNB1 binds to CDK1, stabilizing it and maintaining its kinase activity. In addition to its critical role in G2/M cell cycle transition [16], this complex also phosphorylates multiple downstream targets [17,18]. Previous studies have shown that CDK1 activates IL-6/STAT3 signaling by upregulating GP130 or directly phosphorylating STAT3 [19,20]. However, this mechanism has not been validated in melanoma. To validate this possibility, we performed Co-IP in ME4405 cells and confirmed the direct interaction between CCNB1, CDK1, and STAT3 (Figure 6A), suggesting that CCNB1 stabilizes CDK1 to promote STAT3 activation. Moreover, knockdown of CCNB1 led to a decrease in CDK1 expression, accompanied by a reduction in STAT3 phosphorylation (Figure 6B). Given that STAT3 activation is a key regulator of PD-L1 expression [21,22], we next investigated whether CCNB1 influences PD-L1 levels through the CDK1-STAT3 signaling axis. We confirmed that CCNB1 knockdown reduced PD-L1 at both transcriptional and protein levels in melanoma cells (Figure 6C,D). Furthermore, treatment with RO-3306 led to CCNB1/CDK1 downregulation, accompanied by inhibited STAT3 activation and reduced PD-L1 expression (Figure 6E). Since phosphorylated STAT3 is a known transcriptional activator of IL-6 [19], we next examined IL-6 expression upon CCNB1 modulation. As expected, IL-6 levels decreased in response to CCNB1 knockdown (Figure 6C). RT-qPCR analysis revealed that CCNB1 overexpression upregulated IL-6 transcription (Figure 6F), which was further confirmed by increased IL-6 secretion as measured by ELISA (Figure 6G). Next, we treated NK-92MI cells with conditioned medium from CCNB1-overexpressing ME4405 cells and observed activation of STAT3 signaling (Figure 6H).

To further explore the clinical relevance of our findings, we analyzed immunohistochemical data from the HPA database (the Human Protein Atlas, https://www.proteinatlas.org; accessed on 12 January 2025). Images from two patients, using antibodies from the same source, suggested a possible positive association between CCNB1 and PD-L1 expression, and a potential inverse relationship between CCNB1 and CD56 expression, a marker of NK cell infiltration in humans (Figure 6I). Our findings highlighted that CCNB1 enables melanoma cells to evade NK cell-mediated immunity by activating the CDK1-STAT3 signaling axis, leading to increased IL-6 and PD-L1 expression.

### 2.7. Cell–Cell Communication Reveals TGF-β Signaling in CCNB1-High Melanoma Linked to EMT

**Figure 7:**
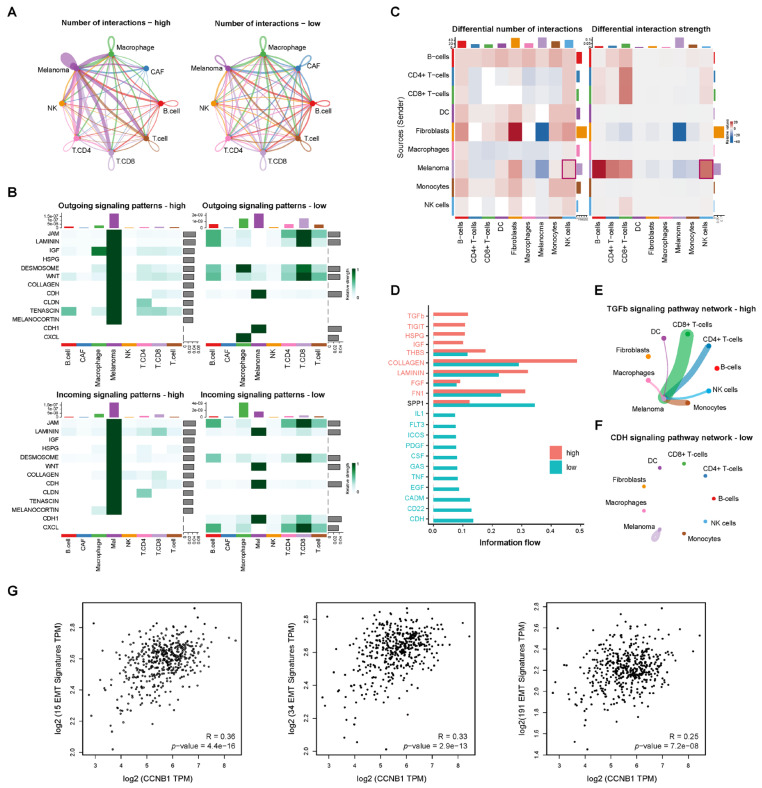
Cell–cell communication reveals TGF-β signaling in CCNB1-high melanoma linked to EMT. (**A**) Network diagrams showing the number of interactions between melanoma cells and various immune and stromal cells, GSE115978 dataset. (**B**) Heatmaps illustrating differences in outgoing (top) and incoming (bottom) signaling patterns between CCNB1-high and CCNB1-low melanoma groups. Key EMT-related signaling pathways, including CDH, CDH1, WNT, Laminin, Collagen, and Tenascin, GSE115978 dataset. (**C**) Heatmaps showing differences in interaction number (left) and interaction strength (right) between immune and stromal cell populations in CCNB1-high and CCNB1-low melanoma groups, GSE189889 dataset. (**D**) Bar graph displaying the top signaling pathways with differential activity between the two groups, GSE189889 dataset. (**E**,**F**) Network diagram showing the TGF-β signaling pathway in CCNB1-high melanoma and CDH signaling pathway in CCNB1-low melanoma, GSE189889 dataset. The color of the lines corresponds to the cell type of the signaling output (indicating directionality), and the width of the lines represents the strength of the signaling. (**G**) Spearman correlation analysis between CCNB1 expression and EMT-related gene signatures from the MSigDB (KOHN_EMT_MESENCHYMAL, ALONSO_METASTASIS_EMT_UP, and FOROUTAN_TGFB_EMT_UP) in the TCGA-SKCM cohort using the GEPIA2 database.

To better understand the role of CCNB1-high melanoma cells in the tumor microenvironment (TME), we applied CellChat, a machine-learning-based method, to analyze single-cell RNA-seq datasets (GSE115978 and GSE189889). Based on CCNB1 expression levels, melanoma cells were classified into high and low CCNB1 groups to explore interaction patterns with immune and stromal cells. In the GSE115978 dataset, melanoma cells with high CCNB1 expression exhibited enhanced cell–cell communication, particularly stronger interactions with NK cells, T cells, macrophages, and CAFs (Figure 7A, left). In contrast, low CCNB1 expression was associated with reduced overall communication (Figure 7A, right). Additionally, EMT-related signaling pathways, including CDH, CDH1, WNT, Laminin, Collagen, and Tenascin, were more active in CCNB1-high melanoma, with both incoming and outgoing signaling interactions strengthened, whereas these signals weakened in CCNB1-low melanoma (Figure 7B). In the GSE189889 dataset, we observed that CCNB1 expression levels correlated with melanoma–NK cell interactions (Figure 7C). Specifically, TGF-β emerged as the dominant signaling pathway in CCNB1-high melanoma (Figure 7D), with network analysis revealing that these cells received stronger TGF-β signals from various sources, including NK cells (Figure 7E). Conversely, in CCNB1-low melanoma, melanoma cells exhibited enhanced intrinsic CDH signaling (Figure 7F).

To further investigate the association between CCNB1 and EMT, we analyzed three EMT-related gene signatures from the MSigDB (KOHN_EMT_MESENCHYMAL, ALONSO_METASTASIS_EMT_UP, and FOROUTAN_TGFB_EMT_UP). Correlation analysis in the GEPIA2 database (http://gepia2.cancer-pku.cn/ (accessed on 15 February 2025), TCGA-SKCM cohort) confirmed a positive correlation between CCNB1 expression and EMT-related gene signatures (Figure 7G). These findings suggested that CCNB1-high melanoma cells might influence melanoma–NK cell interactions while also promoting EMT by enhancing TGF-β signaling in the tumor microenvironment.

### 2.8. CCNB1 Promotes EMT and Melanoma Invasiveness Through the TGF-β-SMAD2/3 Pathway

**Figure 8:**
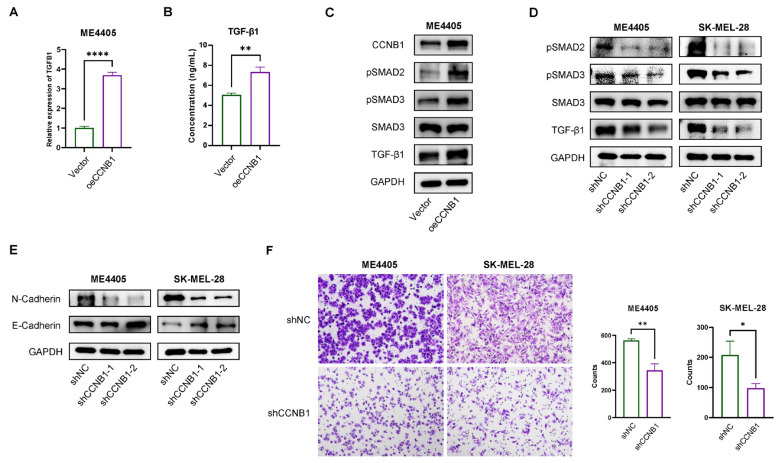
CCNB1 promotes EMT and melanoma invasiveness through the TGF-β-SMAD2/3 pathway. (**A**) TGFB1 mRNA levels in ME4405 cells overexpressing CCNB1 were measured using RT-qPCR. (**B**) ELISA analysis of TGF-β1 levels in the supernatant of ME4405 cells overexpressing CCNB1. (**C**) Western blot analysis showing the effects of CCNB1 overexpression on TGF-β-SMAD2/3 signaling pathway in ME4405. (**D**) Western blot analysis showing the effects of CCNB1 knockdown on TGF-β-SMAD2/3 signaling pathway in ME4405 and SK-MEL-28. (**E**) Western blot analysis showing the impact of CCNB1 knockdown on EMT markers in ME4405 and SK-MEL-28. (**F**) Transwell invasion assay showing the invasive potential of ME4405 and SK-MEL-28 with CCNB1 knockdown. All data are presented as mean ± s.d. Two-sided Student’s *t*-test was used for statistical analysis. * *p* < 0.05, ** *p* < 0.01 and **** *p* < 0.0001. *n* = 3 per group.

To validate our cell–cell communication analysis, an accurate in vitro model replicating NK cell-derived TGF-β signaling within the complex tumor microenvironment is required. However, establishing such a model remains challenging. To our surprise, we found that TGFB1 transcription was increased in CCNB1-overexpressing ME4405 cells (Figure 8A), with ELISA further confirming a significant elevation of secreted TGF-β1 in the supernatant (Figure 8B). Western blot analysis revealed increased TGF-β1 levels and activation of the SMAD2/3 pathway in CCNB1-overexpressing melanoma cells (Figure 8C), whereas CCNB1 knockdown led to reduced TGF-β1 expression and inhibition of SMAD2/3 signaling (Figure 8D). Since the TGF-β-SMAD2/3 pathway is a well-established EMT activator, we next examined key EMT markers, E-Cadherin and N-Cadherin [23]. CCNB1 knockdown led to decreased N-Cadherin levels and increased E-Cadherin, consistent with the inhibition of EMT signaling (Figure 8E). Finally, Transwell invasion assays demonstrated that silencing CCNB1 significantly reduced melanoma cell invasiveness (Figure 8F). These findings highlight CCNB1 as a key driver of melanoma invasiveness, promoting EMT through activation of the TGF-β-SMAD2/3 signaling axis.

## 3. Discussion

**Figure 9:**
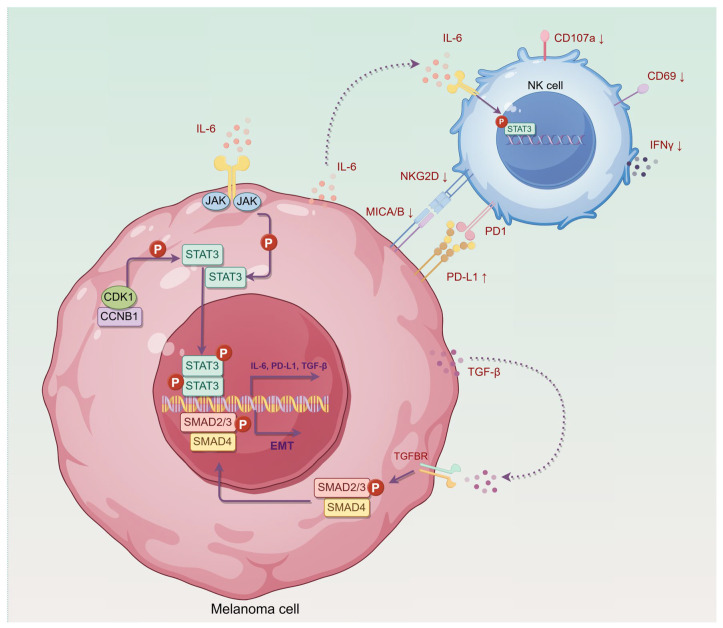
Schematic diagram illustrating the mechanism of CCNB1 in melanoma cells resisting NK cell-mediated cytotoxicity. CCNB1, in complex with CDK1, leads to the phosphorylation and activation of STAT3. Activated STAT3 translocates to the nucleus, promoting the transcription of immune-suppressive molecules such as IL-6 and PD-L1, thereby inhibiting NK cell activity, as evidenced by the downregulation of CD69 and CD107a and reduced IFN-γ secretion. In addition, melanoma-derived IL-6 acts on NK cells to activate STAT3 signaling, which mediates NK cell dysfunction, including NKG2D downregulation. Furthermore, CCNB1 promotes the production of TGF-β in melanoma cells, which activates the SMAD2/3 pathway, thereby promoting the expression of EMT-related genes and enhancing melanoma invasiveness. By Figdraw.

In this study, we provide the first direct evidence that CCNB1 enhances melanoma cell resistance to NK cell-mediated cytotoxicity (Figure 9, summary diagram). We first established a prognostic gene signature for NK cell activation and identified CCNB1 as a druggable target with clinical potential. In vitro, we confirmed that the CCNB1/CDK1 inhibitor RO-3306 suppresses melanoma growth while boosting NK cell infiltration and activation. Mechanistically, we found that CCNB1 stabilizes CDK1 kinase activity, which subsequently phosphorylates STAT3, promoting the transcription of IL-6 and PD-L1. This leads to positive feedback activation of the JAK-STAT3 pathway, upregulation of PD-L1 expression, and suppression of NK cell activation. Additionally, we demonstrated that CCNB1 promotes melanoma EMT and invasiveness through the TGF-β-SMAD2/3 signaling. Given the dual oncogenic role of CCNB1 in resisting NK cell-mediated killing and enhancing melanoma invasiveness, we propose that CCNB1/CDK1 inhibitors represent a promising strategy to enhance NK cell-based immunotherapy for melanoma.

NK cell-based immunotherapy holds great promise for cancer treatment [24], but the heterogeneity of the tumor microenvironment leads to variable responses, particularly in melanoma [25,26]. While there is an urgent need for predictive tools to assess NK cell activation in melanoma, such models remain scarce. Recent studies from Cappello et al. have developed proteomics-based approaches to identify protein signatures associated with NK cell-mediated melanoma killing [27]. However, their model primarily focuses on tumor-intrinsic metabolic factors that influence NK cell susceptibility, without addressing the broader immune evasion mechanisms in the tumor microenvironment. In contrast, our study developed a gene-based prognostic model to predict NK cell activation and melanoma prognosis, identifying CCNB1, PRKACB, FCGR2C, and CCL8 as key prognostic genes. Importantly, our model was validated across multiple independent prognosis and immunotherapy cohorts, demonstrating its robustness in predicting both melanoma prognosis and response to immunotherapy. Our gene signature approach provides an accessible tool that not only predicts NK cell activation but also enables further mechanistic exploration of immune evasion pathways that influence NK cell function in melanoma.

Beyond predicting NK cell activation, we identified CCNB1 as a druggable target with clinical potential and selected it for further investigation, as specific inhibitors are already available. RO-3306, a CCNB1/CDK1 inhibitor, can simultaneously inhibit the activity of both CCNB1 and CDK1 [14], and has previously been shown to suppress tumor-initiating capacity in melanoma by disrupting CDK1-Sox2 signaling [15]. However, it has never been investigated in the context of NK cell activation or tumor immune regulation, leaving its potential role in modulating anti-tumor immunity unexplored. Our study is the first to demonstrate in vivo that RO-3306 suppresses melanoma growth by enhancing NK cell infiltration and increasing the cytotoxic activity of both NK cells and CD8^+^ T cells. In fact, cyclin-dependent kinases (CDKs) have long been recognized as promising therapeutic targets in cancer [28,29]. The development and clinical application of CDK4/6 inhibitors is one of the typical examples [30,31,32]. In melanoma, CDK4/6 inhibitors not only suppress tumor growth but also enhance anti-tumor immunity [33]. Similarly, CCNB1/CDK1 has been identified as a key therapeutic target in multiple cancers, and targeted inhibitors against this pathway are actively being developed [34,35].

To further validate the specific role of CCNB1 in driving NK cell dysfunction in melanoma, we used NK-92MI cells, an IL-2-independent NK cell line widely used in studies on NK cell therapy and immune tolerance [36,37,38,39]. Specifically, we evaluated CD69 as an activation marker, CD107a as a degranulation marker, and IFN-γ as a key cytotoxic effector. Co-culturing NK-92MI cells with CCNB1-knockdown melanoma cells led to a significant increase in all three markers, indicating enhanced NK cell activation, degranulation, and cytotoxicity. Additionally, we examined the MICA/MICB-NKG2D axis, a crucial mechanism for NK cell-mediated tumor recognition and killing [40], and found that CCNB1-high melanoma cells suppress its activation. Our findings suggest that CCNB1 expression in melanoma cells may impair NK cell cytotoxicity by modulating both intrinsic tumor properties and NK cell activation status. These results align with previous studies showing that tumor cells exploit immune checkpoint pathways and stress-induced ligand regulation to evade NK cell surveillance [25].

Given these findings, we sought to understand the molecular mechanisms by which CCNB1 promotes NK cell resistance in melanoma. CCNB1 is a key cell cycle regulator that binds to CDK1 to drive the G2/M transition [16]. Beyond this well-known role, the CCNB1/CDK1 complex also influences tumor-related signaling pathways [15,41]. Studies have shown that CDK1 promotes IL-6 receptor GP130 expression or indirectly phosphorylates STAT3, activating the JAK-STAT3 pathway [18]. Consistent with this, our Co-IP experiments confirmed a direct interaction between CCNB1/CDK1 and STAT3, suggesting a potential mechanism for direct STAT3 activation. Furthermore, we demonstrated that RO-3306 reduces STAT3 phosphorylation by inhibiting CCNB1/CDK1, aligning with the findings of Chen et al. in diffuse large B-cell lymphoma [42]. Since STAT3 activation is sustained by IL-6 signaling, CCNB1 likely contributes to a positive feedback loop between IL-6 and JAK-STAT3, further reinforcing tumor immune evasion. Notably, IL-6 secreted by CCNB1-high melanoma cells not only maintains STAT3 activation within tumor cells but also enhances STAT3 signaling in NK-92MI cells, potentially exacerbating NK cell dysfunction [43]. Additionally, PD-L1 is a key immune checkpoint that suppresses anti-tumor immunity and is transcriptionally regulated by JAK-STAT3 signaling [22]. Our findings indicate that CCNB1 enhances PD-L1 expression, further contributing to immune escape. These findings reveal that CCNB1/CDK1 drives melanoma immune evasion by activating IL-6/STAT3/PD-L1 signaling and promoting NK cell dysfunction.

Our cell–cell communication analysis revealed that CCNB1-high melanoma cells may significantly impact interactions with the tumor microenvironment, particularly with NK cells, which can transmit TGF-β signals to melanoma cells and influence EMT. However, replicating and validating this phenomenon in vitro remains challenging. In fact, previous studies have reported that NK cells can promote tumor metastasis and progression by transmitting signals to tumor cells [44,45]. Specifically, activation of the STAT3 pathway within NK cells enhances TGF-β secretion, which in turn induces EMT and a metastatic phenotype in tumor cells [43]. Our results demonstrated that CCNB1-high melanoma cells secrete elevated IL-6, a known activator of STAT3 signaling in NK cells [46]. Indeed, treating NK-92MI cells with supernatant from CCNB1-overexpressing melanoma cells activated STAT3 signaling. This suggests that CCNB1-driven IL-6 secretion may stimulate NK cells to release pro-invasive signals such as TGF-β, which in turn promotes melanoma invasiveness. Additionally, we found that CCNB1 directly enhances TGF-β production and secretion in melanoma cells, activating the SMAD2/3 pathway to drive EMT and tumor invasion. Beyond its role in tumor progression, TGF-β is a well-known immunosuppressive factor, impairing NK cell function by downregulating NKG2D expression and inhibiting IFN-γ production [47,48], which was also observed in our study. Overall, the interaction between CCNB1-high melanoma cells and NK cells in the tumor microenvironment is complex, requiring further studies to validate key mechanisms.

Despite these important findings, our study has several limitations. First, we primarily focused on how CCNB1 mediates immune tolerance in melanoma cells but did not investigate how the upregulated TGF-β affect NK cells and other immune cells in the tumor microenvironment. Second, the anti-tumor effects of CCNB1/CDK1 inhibition extend beyond PD-L1 regulation, including modulation of the IL-6-JAK-STAT3 axis and TGF-β signaling. Further studies are needed to explore the potential synergy between CCNB1/CDK1 inhibitors and anti-PD-1/PD-L1 checkpoint blockade. Finally, CCNB1/CDK1 inhibitors are still in the preclinical research phase, and the development of more specific inhibitors along with comprehensive safety evaluations are urgently needed.

## 4. Materials and Methods

### 4.1. Data Source

RNA sequencing (RNA-seq) and clinical data from patients were obtained from three major public repositories: The Cancer Genome Atlas (TCGA) (https://www.cancer.gov/tcga; accessed on 8 September 2024), Gene Expression Omnibus (GEO) (https://www.ncbi.nlm.nih.gov/geo; accessed on 8 September 2024), and cBioPortal (https://www.cbioportal.org; accessed on 8 September 2024). The datasets used in this study consist of seven previously published melanoma cohorts: TCGA-SKCM (*n* = 455), GSE59455 (*n* = 122), GSE65904 (*n* = 210), GSE54467 (*n* = 78), GSE115978 (*n* = 31), GSE189889 (*n* = 9), and the DFCI cohort (*n* = 121). Data for the DFCI cohort were downloaded from the cBioPortal public database [49].

### 4.2. Development NK Activation-Related Prognostic Model

The TCGA-SKCM cohort was used as the training dataset to construct the model. CIBERSORT estimated the relative abundance of 22 immune cell types, stratifying patients into high and low infiltration groups based on the median infiltration level [50]. Kaplan–Meier survival analysis assessed survival differences. WGCNA identified co-expression modules among NK activation-related genes, selecting a soft threshold for network construction and determining the most relevant module [51]. Univariate Cox regression identified survival-associated genes, which were clustered using ConsensusClusterPlus, yielding two clusters validated by consensus matrix and tree analysis [52]. Differentially expressed genes between clusters were identified with the limma package [53], and survival-related genes were further confirmed through univariate Cox regression. Intersecting DEGs with Innate Immunity genes from GeneCards (https://www.genecards.org/; accessed on 12 September 2024) resulted in NKAGs. LASSO regression minimized overfitting, and bidirectional stepwise regression refined the final Cox model, selecting the optimal prognostic genes based on AIC minimization [54].

### 4.3. Gene Set Variation Analysis

Gene Set Variation Analysis (GSVA) combined with differential analysis was used to compare biological pathway activities between high- and low-risk groups [55]. The “GSVA” R package calculated activity scores for C2 pathway gene sets from MSigDB (https://www.gsea-msigdb.org/gsea/msigdb; accessed on 15 September 2024), covering diverse signaling pathways. Differential analysis using “limma” identified significantly altered pathways, categorized based on t-values: positive values indicated higher activity in the high-risk group, while negative values reflected greater activity in the low-risk group.

### 4.4. Cell–Cell Communication Analysis

The “CellChat” R package, referencing CellChatDB.human (http://www.cellchat.org/; accessed on 8 November 2024) for known ligands, receptors, and cofactors, was used to analyze the number and strength of cell–cell interactions in the GSE115978 and GSE189889 single-cell RNA-sequencing dataset. Samples were grouped into high and low categories based on the median expression level of CCNB1 in melanoma cells. Heatmaps illustrated differences in ligand–receptor pair interactions across cell types between the two groups, showing overall signaling patterns. Specific signals were further visualized in network diagrams to depict interactions between melanoma cells and immune cells.

### 4.5. Cell Culture and Stable Cell Line Generation

The natural killer cell line NK-92MI (RRID: CVCL_3755) was cultured in complete medium (TAKARA, GT-T551 H3), with no additional components required. ME4405 (RRID: CVCL_C680) was cultured in DMEM medium, and SK-MEL-28 (RRID: CVCL_0526) cells were cultured in RPMI-1640 medium, both supplemented with 10% fetal bovine serum (FBS) and incubated at 37 °C in a 5% CO_2_ atmosphere. Lentiviral pLKO.1-shCCNB1-puro (constructed in-house) and pLV3-oeCCNB1-puro (Miaoling Biotechnology, Wuhan, China) were used to transduce ME4405 and SK-MEL-28 melanoma cells. Lentiviral particles were generated by PEI-mediated transfection of 293T cells with the respective plasmids and packaging vectors. After 48 h transduction, cells were selected with puromycin (2 µg/mL, 72 h) to establish stable lines. Western blotting confirmed CCNB1 knockdown or overexpression.

### 4.6. Co-Culture of NK-92MI Cells with Melanoma Cells and Assessment by Flow Cytometry

For NK cell activation marker detection, NK-92MI cells were co-cultured with melanoma cells at an effector-to-target (E:T) ratio of 10:1 for 12 h. Cells were stained for viability using BD Horizon™ Fixable Viability Stain 510 (BD Biosciences, San Jose, CA, USA, Cat No. 564406) and surface markers CD56 (BD Biosciences, San Jose, CA, USA, Cat No. 564057), CD69 (BD Biosciences, San Jose, CA, USA, Cat No. 560738), CD107a (BD Biosciences, San Jose, CA, USA, Cat No. 561348), MICA/MICB (Biolegend, San Diego, CA, USA, Cat No. 320907), and NKG2D (Biolegend, San Diego, CA, USA, Cat No. 320817). Staining was performed on ice for 30 min in the dark, followed by washing. For IFN-γ detection, Cell Stimulation Cocktail (BD Biosciences, San Jose, CA, USA, Cat No. 555028) was used for 4 h incubation at 37 °C, followed by intracellular staining with IFN-γ antibody (Biolegend, San Diego, CA, USA, Cat No. 502520). For NK cell cytotoxicity assessment, melanoma and NK-92MI cells were co-cultured as described above. Apoptosis was analyzed using Annexin V/PI apoptosis kit (MultiSciences Biotech, Hangzhou, China, Cat No. AP107), staining for 5 min at room temperature in the dark. Samples were analyzed within 1 h using CytoFLEX LX Flow Cytometer (Beckman Coulter, Brea, CA, USA), and apoptotic tumor cells (CD56^−^) were identified using FlowJo software (version 10.8.1), categorizing early (Annexin V^+^/PI^−^) and late (Annexin V^+^/PI^+^) apoptosis. Apoptosis rates were statistically compared under different conditions.

### 4.7. Western Blotting and Co-Immunoprecipitation (Co-IP)

Cells were lysed in protein extraction buffer, and the lysates were used for SDS-PAGE and immunoblot analysis. The following are the primary antibodies used in this study: CCNB1 (1:2000; Proteintech, Wuhan, China, Cat No. 55004-1-AP), CDK1 (1:2000; Proteintech, Wuhan, China, Cat No. 19532-1-AP), STAT3 (1:1000; Cell Signaling Technology, Danvers, MA, USA, Cat No. 9139S), phospho-STAT3 (Tyr705) (1:1000; Cell Signaling Technology, Danvers, MA, USA, Cat No. 4113S), IL-6 (1:2000; Proteintech, Wuhan, China, Cat No. 66146-1-Ig), PD-L1 (1:1000; GeneTex, San Antonio, TX, USA, Cat No. GTX104763), phospho-SMAD2 (ser465/467) (1:1000; Cell Signaling Technology, Danvers, MA, USA, Cat No. 3101), SMAD3 (1:1000; Cell Signaling Technology, Danvers, MA, USA, Cat No. 9513), phospho-SMAD3 (Ser423/425) (1:1000; Cell Signaling Technology, Danvers, MA, USA, Cat No. 9520), TGF-β1 (1:2000; HUABIO, Hangzhou, China, Cat No. HA721143), E-cadherin (1:5000; Proteintech, Wuhan, China, Cat No. 20874-1-AP), N-cadherin (1:2000; Proteintech, Wuhan, China, Cat No. 22018-1-AP), GAPDH (1:1000; Cell Signaling Technology, Danvers, MA, USA, Cat No. 2118). Stable Flag-CCNB1-transfected ME4405 melanoma cells were subjected to Co-IP experiments, with untransfected control cells used as a comparison. Cells were lysed on ice using RIPA buffer, and the lysates were incubated overnight at 4 °C with Flag-tagged immunomagnetic beads (Targetmol, Shanghai, China, C0120B). After incubation, the beads were washed with PBS to remove nonspecifically bound proteins. The Co-IP products and input control samples were separated by SDS-PAGE and analyzed by Western blotting.

### 4.8. RT-qPCR

Total mRNAs from cells were extracted with the EZ-press RNA Purification Kit (EZBioscience, Fairview Ave, Roseville, CA, USA, B0004D) and reverse-transcribed using the Reverse Transcription Kit (Takara, Kusatsu, Shiga, Japan, RR047A). Fluorescence quantitative PCR was performed with ChamQ Universal SYBR qPCR MasterMIx (Vazyme, Jiangsu, China, Q711-02). The primer sequences used for RT-qPCR are listed in Supplementary Table S1.

### 4.9. Enzyme-Linked Immunosorbent Assay (ELISA)

Cells were seeded in 6-well plates and cultured to 70–80% confluency before replacing the medium with serum-free culture medium for 48 h at 37 °C with 5% CO_2_. The supernatant was collected for ELISA analysis of IL-6 and TGF-β1. For TGF-β1 detection, activation was performed by incubating with 1N HCl for 10 min, followed by neutralization with 1 N NaOH. IL-6 and TGF-β1 levels were measured using ELISA kits (Ruixin Biotech, Quanzhou, China, RX106126H and RX104768H) according to the manufacturer’s protocol. Absorbance at 450 nm was recorded using a BioTek Epoch microplate reader. To correct for baseline TGF-β1 levels in the medium, a cell-free control was included, and its value was subtracted from all readings. Standard curves were generated using ELISA Calc software.

### 4.10. Cell Invasion Assay

Cell invasion was evaluated using Transwell plates (Corning, Cat No. 353097) with an 8 μm pore size, where the upper chamber was pre-coated with Matrigel (Corning, Cat No. 354234) to mimic the extracellular matrix. To prepare the coating, 100 μL of Matrigel was added to the upper chamber surface and incubated at 37 °C for 1 h to ensure stability. Melanoma cells were then suspended in serum-free medium and seeded into the upper chamber at a density of 2 × 10^5^ cells per well, while the lower chamber was filled with 600 μL of medium containing 20% FBS. After 48 h of incubation, non-invasive cells on the upper surface were gently removed with a cotton swab. The invading cells on the underside of the membrane were stained with 0.1% crystal violet, and the number of invading cells was counted. ImageJ software (version 1.54) was used to analyze the results and assess cell invasion capacity.

### 4.11. Animal Models and Treatment

To establish a subcutaneous melanoma model, 1 × 10^5^ B16-F10 cells were resuspended in 100 µL phosphate-buffered saline (PBS) and subcutaneously injected into the flank of 6- to 7-week-old C57BL/6 mice. On day 6 post-inoculation, mice (*n* = 5 per group) were randomly assigned to receive RO-3306 (4 mg/kg, Targetmol, Shanghai, China, T2356) every three days, or PBS as a control. Tumor growth was monitored every three days, and tumor volume was calculated as length × width^2^ × 0.5. At the end of treatment, mice were euthanized, and tumor single-cell suspensions were prepared for flow cytometry analysis.

### 4.12. Statistical Analysis

Statistical analyses were conducted using GraphPad Prism (Version 9, GraphPad) and R software (version 4.3.3). The Shapiro–Wilk test was used to evaluate data normality, while the F-test assessed variance homogeneity. For comparisons between two groups that met the assumptions of normality and equal variances, an unpaired two-tailed *t*-test was used to analyze differences in means. If the data did not follow a normality, the Mann–Whitney U test was applied as a non-parametric alternative. If the assumption of equal variances was not met, Welch’s corrected unpaired *t*-test was used. Results are presented as the mean ± standard deviation (s.d.), with a *p*-value < 0.05 considered statistically significant.

## 5. Conclusions

This study highlights the critical role of CCNB1/CDK1 in melanoma resistance to NK cell-mediated cytotoxicity and tumor invasiveness. We demonstrate that CCNB1/CDK1 promotes immune evasion by activating the JAK-STAT3 pathway, leading to upregulation of IL-6, and PD-L1, which suppress NK cell function. Additionally, CCNB1 activates the TGF-β-SMAD2/3 pathway, driving EMT and enhancing melanoma invasiveness. These dual effects suggest that CCNB1/CDK1 is a promising therapeutic target in melanoma and warrants further investigation.

## Data Availability

The datasets used in this study are publicly available. RNA sequencing and clinical data were obtained from The Cancer Genome Atlas (TCGA) (https://www.cancer.gov/tcga; accessed on 8 September 2024) and the Gene Expression Omnibus (GEO) (https://www.ncbi.nlm.nih.gov/geo; accessed on 8 September 2024). The melanoma cohorts analyzed include TCGA-SKCM, GSE59455, GSE65904, GSE54467, GSE115978, and GSE189889, which were downloaded from TCGA and GEO, while data for the DFCI cohort were retrieved from the cBioPortal database (https://www.cbioportal.org; accessed on 8 September 2024). Immunohistochemical images of melanoma tissue analyzed in this study were obtained from the Human Protein Atlas (HPA) (https://www.proteinatlas.org; accessed on 12 January 2025). All these datasets are freely accessible from their respective repositories.

## Supplementary Materials

The following supporting information can be downloaded at https://www.mdpi.com/article/10.3390/ph18050666/s1, Figure S1. Analysis of gene co-expression networks and feature selection for predictive modeling. (A) Determination of the soft-thresholding power in WGCNA. (B) Gene dendrogram and module identification using WGCNA. (C,D) Consensus clustering analysis for stratifying samples into subgroups. (E) Bar plot showing the final consensus clustering results, confirming that the samples were divided into two stable clusters. (F) LASSO coefficient profiles. (G) Selection of optimal λ in LASSO; Figure S2. Receiver operating characteristic (ROC) curves for predicting patient survival across multiple cohorts. (A–D) ROC curves evaluating 1-, 2-, 3-, and 5-year overall survival (OS) predictions for the constructed model across training and validation cohorts (TCGA-SKCM, GSE59455, GSE65904,GSE54467). (E) Anti-PD-1 immune checkpoint blockade cohort (DFCI, Nature Medicine 2019) shows Area Under the Curve (AUC) values for 1-, 2-, and 3-year OS, respectively; Table S1. Real-time qPCR primers used in this research.

## Abbreviations

NK: Natural killer; CDKs: Cyclin-dependent kinases; TME: Tumor microenvironment; WGCNA: Weighted gene co-expression network analysis; LASSO: Least absolute shrinkage and selection operator; TCGA: The cancer genome atlas; SKCM: Skin cutaneous melanoma; IFN-γ: Interferon-gamma; NKG2D: NK group 2 member D; MICA/B: MHC class I chain-related protein A and B; IL-6: Interleukin 6; TGF-β: Transforming growth factor-β; PD-L1: Programmed death ligand 1; EMT: Epithelial–mesenchymal transition; MHC-I: Major histocompatibility complex I; GSVA: Gene set variation analysis; scRNA-Seq: single-cell RNA sequencing; OS: Overall survival; Co-IP: Co-immunoprecipitation; RT-qPCR: Real-time quantitative polymerase chain reaction; ICB: Immune checkpoint blockade.

## References

[1] Shain A.H., Bastian B.C. (2016). From Melanocytes to Melanomas. Nat. Rev. Cancer.

[2] Long G.V., Swetter S.M., Menzies A.M., Gershenwald J.E., Scolyer R.A. (2023). Cutaneous Melanoma. Lancet Lond. Engl..

[3] Berk-Krauss J., Stein J.A., Weber J., Polsky D., Geller A.C. (2020). New Systematic Therapies and Trends in Cutaneous Melanoma Deaths Among US Whites, 1986–2016. Am. J. Public Health.

[4] Patel S.P., Othus M., Chen Y., Wright G.P., Yost K.J., Hyngstrom J.R., Hu-Lieskovan S., Lao C.D., Fecher L.A., Truong T.-G. (2023). Neoadjuvant-Adjuvant or Adjuvant-Only Pembrolizumab in Advanced Melanoma. N. Engl. J. Med..

[5] Long G.V., Carlino M.S., McNeil C., Ribas A., Gaudy-Marqueste C., Schachter J., Nyakas M., Kee D., Petrella T.M., Blaustein A. (2024). Pembrolizumab versus Ipilimumab for Advanced Melanoma: 10-Year Follow-up of the Phase III KEYNOTE-006 Study. Ann. Oncol. Off. J. Eur. Soc. Med. Oncol..

[6] Huang A.C., Zappasodi R. (2022). A Decade of Checkpoint Blockade Immunotherapy in Melanoma: Understanding the Molecular Basis for Immune Sensitivity and Resistance. Nat. Immunol..

[7] Ziogas D.C., Theocharopoulos C., Koutouratsas T., Haanen J., Gogas H. (2023). Mechanisms of Resistance to Immune Checkpoint Inhibitors in Melanoma: What We Have to Overcome?. Cancer Treat. Rev..

[8] Wolf N.K., Kissiov D.U., Raulet D.H. (2023). Roles of Natural Killer Cells in Immunity to Cancer, and Applications to Immunotherapy. Nat. Rev. Immunol..

[9] Delconte R.B., Kolesnik T.B., Dagley L.F., Rautela J., Shi W., Putz E.M., Stannard K., Zhang J.-G., Teh C., Firth M. (2016). CIS Is a Potent Checkpoint in NK Cell-Mediated Tumor Immunity. Nat. Immunol..

[10] Raja R., Wu C., Bassoy E.Y., Rubino T.E., Utagawa E.C., Magtibay P.M., Butler K.A., Curtis M. (2022). PP4 Inhibition Sensitizes Ovarian Cancer to NK Cell-Mediated Cytotoxicity via STAT1 Activation and Inflammatory Signaling. J. Immunother. Cancer.

[11] Page A., Chuvin N., Valladeau-Guilemond J., Depil S. (2024). Development of NK Cell-Based Cancer Immunotherapies through Receptor Engineering. Cell. Mol. Immunol..

[12] Davis-Marcisak E.F., Fitzgerald A.A., Kessler M.D., Danilova L., Jaffee E.M., Zaidi N., Weiner L.M., Fertig E.J. (2021). Transfer Learning between Preclinical Models and Human Tumors Identifies a Conserved NK Cell Activation Signature in Anti-CTLA-4 Responsive Tumors. Genome Med..

[13] Pan R., Ryan J., Pan D., Wucherpfennig K.W., Letai A. (2022). Augmenting NK Cell-Based Immunotherapy by Targeting Mitochondrial Apoptosis. Cell.

[14] Vassilev L.T., Tovar C., Chen S., Knezevic D., Zhao X., Sun H., Heimbrook D.C., Chen L. (2006). Selective Small-Molecule Inhibitor Reveals Critical Mitotic Functions of Human CDK1. Proc. Natl. Acad. Sci. USA.

[15] Ravindran Menon D., Luo Y., Arcaroli J.J., Liu S., KrishnanKutty L.N., Osborne D.G., Li Y., Samson J.M., Bagby S., Tan A.-C. (2018). CDK1 Interacts with Sox2 and Promotes Tumor Initiation in Human Melanoma. Cancer Res..

[16] Wang Z., Fan M., Candas D., Zhang T.-Q., Qin L., Eldridge A., Wachsmann-Hogiu S., Ahmed K.M., Chromy B.A., Nantajit D. (2014). Cyclin B1/Cdk1 Coordinates Mitochondrial Respiration for Cell-Cycle G2/M Progression. Dev. Cell.

[17] Liu P., Kao T.P., Huang H. (2008). CDK1 Promotes Cell Proliferation and Survival via Phosphorylation and Inhibition of FOXO1 Transcription Factor. Oncogene.

[18] Lu M., Breyssens H., Salter V., Zhong S., Hu Y., Baer C., Ratnayaka I., Sullivan A., Brown N.R., Endicott J. (2013). Restoring P53 Function in Human Melanoma Cells by Inhibiting MDM2 and Cyclin B1/CDK1-Phosphorylated Nuclear iASPP. Cancer Cell.

[19] Kuang Y., Guo W., Ling J., Xu D., Liao Y., Zhao H., Du X., Wang H., Xu M., Song H. (2019). Iron-Dependent CDK1 Activity Promotes Lung Carcinogenesis via Activation of the GP130/STAT3 Signaling Pathway. Cell Death Dis..

[20] Xu X., Ding Y., Jin J., Xu C., Hu W., Wu S., Ding G., Cheng R., Cao L., Jia S. (2023). Post-Translational Modification of CDK1-STAT3 Signaling by Fisetin Suppresses Pancreatic Cancer Stem Cell Properties. Cell Biosci..

[21] Johnson D.E., O’Keefe R.A., Grandis J.R. (2018). Targeting the IL-6/JAK/STAT3 Signalling Axis in Cancer. Nat. Rev. Clin. Oncol..

[22] Bu L.L., Yu G.T., Wu L., Mao L., Deng W.W., Liu J.F., Kulkarni A.B., Zhang W.F., Zhang L., Sun Z.J. (2017). STAT3 Induces Immunosuppression by Upregulating PD-1/PD-L1 in HNSCC. J. Dent. Res..

[23] Xu J., Lamouille S., Derynck R. (2009). TGF-Beta-Induced Epithelial to Mesenchymal Transition. Cell Res..

[24] Peng L., Sferruzza G., Yang L., Zhou L., Chen S. (2024). CAR-T and CAR-NK as Cellular Cancer Immunotherapy for Solid Tumors. Cell. Mol. Immunol..

[25] Ghaedrahmati F., Esmaeil N., Abbaspour M. (2023). Targeting Immune Checkpoints: How to Use Natural Killer Cells for Fighting against Solid Tumors. Cancer Commun. Lond. Engl..

[26] Zhu L., Kalimuthu S., Gangadaran P., Oh J.M., Lee H.W., Baek S.H., Jeong S.Y., Lee S.-W., Lee J., Ahn B.-C. (2017). Exosomes Derived from Natural Killer Cells Exert Therapeutic Effect in Melanoma. Theranostics.

[27] Cappello S., Sung H.-M., Ickes C., Gibhardt C.S., Vultur A., Bhat H., Hu Z., Brafford P., Denger A., Stejerean-Todoran I. (2021). Protein Signatures of NK Cell-Mediated Melanoma Killing Predict Response to Immunotherapies. Cancer Res..

[28] Asghar U., Witkiewicz A.K., Turner N.C., Knudsen E.S. (2015). The History and Future of Targeting Cyclin-Dependent Kinases in Cancer Therapy. Nat. Rev. Drug Discov..

[29] Bury M., Le Calvé B., Ferbeyre G., Blank V., Lessard F. (2021). New Insights into CDK Regulators: Novel Opportunities for Cancer Therapy. Trends Cell Biol..

[30] Dhillon S. (2015). Palbociclib: First Global Approval. Drugs.

[31] Syed Y.Y. (2017). Ribociclib: First Global Approval. Drugs.

[32] Dhillon S. (2021). Trilaciclib: First Approval. Drugs.

[33] Lelliott E.J., Sheppard K.E., McArthur G.A. (2022). Harnessing the Immunotherapeutic Potential of CDK4/6 Inhibitors in Melanoma: Is Timing Everything?. NPJ Precis. Oncol..

[34] Lu X., Pang Y., Cao H., Liu X., Tu L., Shen Y., Jia X., Lee J.-C., Wang Y. (2021). Integrated Screens Identify CDK1 as a Therapeutic Target in Advanced Gastrointestinal Stromal Tumors. Cancer Res..

[35] Akl L., Abd El-Hafeez A.A., Ibrahim T.M., Salem R., Marzouk H.M.M., El-Domany R.A., Ghosh P., Eldehna W.M., Abou-Seri S.M. (2022). Identification of Novel Piperazine-Tethered Phthalazines as Selective CDK1 Inhibitors Endowed with in Vitro Anticancer Activity toward the Pancreatic Cancer. Eur. J. Med. Chem..

[36] Li S., Mirlekar B., Johnson B.M., Brickey W.J., Wrobel J.A., Yang N., Song D., Entwistle S., Tan X., Deng M. (2022). STING-Induced Regulatory B Cells Compromise NK Function in Cancer Immunity. Nature.

[37] Wu L., Liu F., Yin L., Wang F., Shi H., Zhao Q., Yang F., Chen D., Dong X., Gu Y. (2022). The Establishment of Polypeptide PSMA-Targeted Chimeric Antigen Receptor-Engineered Natural Killer Cells for Castration-Resistant Prostate Cancer and the Induction of Ferroptosis-Related Cell Death. Cancer Commun. Lond. Engl..

[38] Morgan H.J., Rees E., Lanfredini S., Powell K.A., Gore J., Gibbs A., Lovatt C., Davies G.E., Olivero C., Shorning B.Y. (2022). CD200 Ectodomain Shedding into the Tumor Microenvironment Leads to NK Cell Dysfunction and Apoptosis. J. Clin. Investig..

[39] Gong Z., Li Q., Shi J., Liu E.T., Shultz L.D., Ren G. (2022). Lipid-Laden Lung Mesenchymal Cells Foster Breast Cancer Metastasis via Metabolic Reprogramming of Tumor Cells and Natural Killer Cells. Cell Metab..

[40] Ferrari de Andrade L., Tay R.E., Pan D., Luoma A.M., Ito Y., Badrinath S., Tsoucas D., Franz B., May K.F., Harvey C.J. (2018). Antibody-Mediated Inhibition of MICA and MICB Shedding Promotes NK Cell-Driven Tumor Immunity. Science.

[41] Malumbres M., Barbacid M. (2009). Cell Cycle, CDKs and Cancer: A Changing Paradigm. Nat. Rev. Cancer.

[42] Chen Q., Lu C., Li D., Xu L., Wang C., Yu L. (2025). CDK1 Inhibitor RO-3306 Enhances BTKi Potency in Diffuse Large B-Cell Lymphoma by Suppressing JAK2/STAT3 Signaling. Int. J. Biol. Macromol..

[43] Neo S.Y., Yang Y., Record J., Ma R., Chen X., Chen Z., Tobin N.P., Blake E., Seitz C., Thomas R. (2020). CD73 Immune Checkpoint Defines Regulatory NK Cells within the Tumor Microenvironment. J. Clin. Investig..

[44] Neo S.Y., Oliveira M.M.S., Tong L., Chen Y., Chen Z., Cismas S., Burduli N., Malmerfelt A., Teo J.K.H., Lam K.-P. (2024). Natural Killer Cells Drive 4-1BBL Positive Uveal Melanoma towards EMT and Metastatic Disease. J. Exp. Clin. Cancer Res. CR.

[45] Chan I.S., Knútsdóttir H., Ramakrishnan G., Padmanaban V., Warrier M., Ramirez J.C., Dunworth M., Zhang H., Jaffee E.M., Bader J.S. (2020). Cancer Cells Educate Natural Killer Cells to a Metastasis-Promoting Cell State. J. Cell Biol..

[46] Cacalano N.A. (2016). Regulation of Natural Killer Cell Function by STAT3. Front. Immunol..

[47] Donatelli S.S., Zhou J.-M., Gilvary D.L., Eksioglu E.A., Chen X., Cress W.D., Haura E.B., Schabath M.B., Coppola D., Wei S. (2014). TGF-β-Inducible microRNA-183 Silences Tumor-Associated Natural Killer Cells. Proc. Natl. Acad. Sci. USA.

[48] Ng S., Deng J., Chinnadurai R., Yuan S., Pennati A., Galipeau J. (2016). Stimulation of Natural Killer Cell-Mediated Tumor Immunity by an IL15/TGFβ-Neutralizing Fusion Protein. Cancer Res..

[49] Liu D., Schilling B., Liu D., Sucker A., Livingstone E., Jerby-Arnon L., Zimmer L., Gutzmer R., Satzger I., Loquai C. (2019). Integrative Molecular and Clinical Modeling of Clinical Outcomes to PD1 Blockade in Patients with Metastatic Melanoma. Nat. Med..

[50] Newman A.M., Liu C.L., Green M.R., Gentles A.J., Feng W., Xu Y., Hoang C.D., Diehn M., Alizadeh A.A. (2015). Robust Enumeration of Cell Subsets from Tissue Expression Profiles. Nat. Methods.

[51] Langfelder P., Horvath S. (2008). WGCNA: An R Package for Weighted Correlation Network Analysis. BMC Bioinform..

[52] Wilkerson M.D., Hayes D.N. (2010). ConsensusClusterPlus: A Class Discovery Tool with Confidence Assessments and Item Tracking. Bioinform. Oxf. Engl..

[53] Ritchie M.E., Phipson B., Wu D., Hu Y., Law C.W., Shi W., Smyth G.K. (2015). Limma Powers Differential Expression Analyses for RNA-Sequencing and Microarray Studies. Nucleic Acids Res..

[54] Friedman J., Hastie T., Tibshirani R. (2010). Regularization Paths for Generalized Linear Models via Coordinate Descent. J. Stat. Softw..

[55] Hänzelmann S., Castelo R., Guinney J. (2013). GSVA: Gene Set Variation Analysis for Microarray and RNA-Seq Data. BMC Bioinform..

