# Supplementary material for: Natural Killer Cell Activation Signature Identifies Cyclin B1/CDK1 as a Druggable Target to Overcome Natural Killer Cell Dysfunction and Tumor Invasiveness in Melanoma
Source: Pharmaceuticals (Basel). 2025 Apr 30;18(5):666. doi: 10.3390/ph18050666 (PMC12114673; doi:10.3390/ph18050666)
Supplement: Supplementary file 1 [file pharmaceuticals-18-00666-s001.zip › Supplemental Figure 2.pdf]

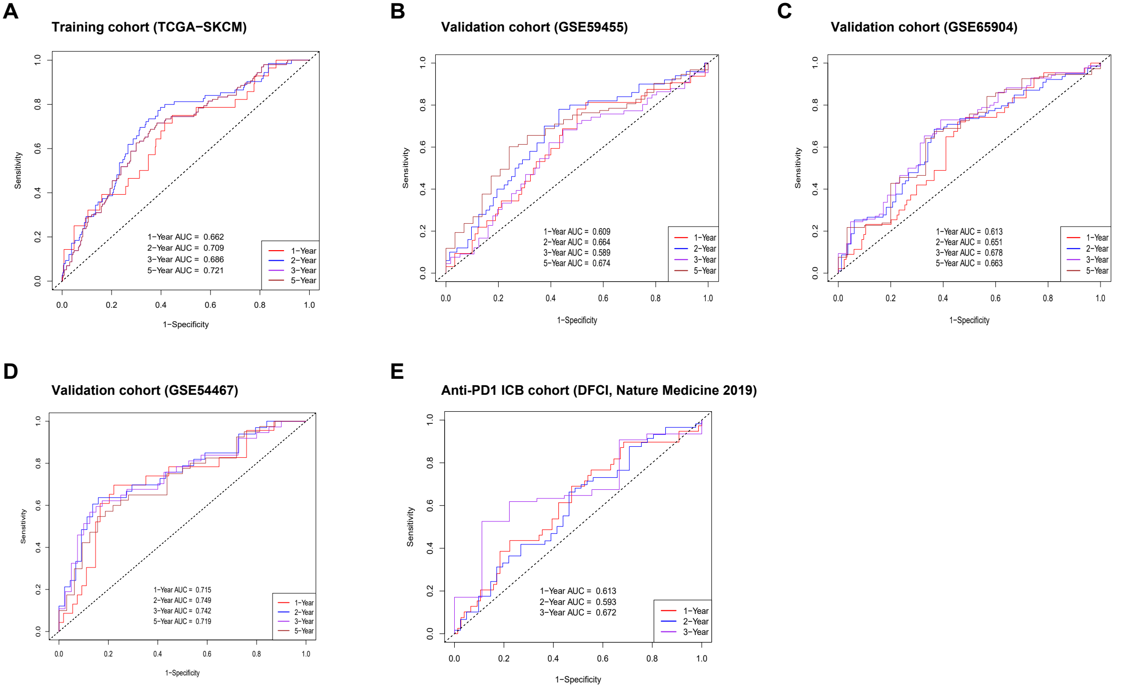
A
Training cohort (TCGA−SKCM)
1−Year AUC = 0.662
2−Year AUC = 0.709
3−Year AUC = 0.686
5−Year AUC = 0.721
1−Year
2−Year
3−Year
5−Year
Sensitivity
1−Specificity
B
Validation cohort (GSE59455)
1−Year AUC = 0.609
2−Year AUC = 0.664
3−Year AUC = 0.589
5−Year AUC = 0.674
1−Year
2−Year
3−Year
5−Year
Sensitivity
1−Specificity
C
Validation cohort (GSE65904)
1−Year AUC = 0.613
2−Year AUC = 0.651
3−Year AUC = 0.678
5−Year AUC = 0.663
1−Year
2−Year
3−Year
5−Year
Sensitivity
1−Specificity
D
Validation cohort (GSE54467)
1−Year AUC = 0.715
2−Year AUC = 0.749
3−Year AUC = 0.742
5−Year AUC = 0.719
1−Year
2−Year
3−Year
5−Year
Sensitivity
1−Specificity
E
Anti-PD1 ICB cohort (DFCI, Nature Medicine 2019)
1−Year AUC = 0.613
2−Year AUC = 0.593
3−Year AUC = 0.672
1−Year
2−Year
3−Year
Sensitivity
1−Specificity
