# Supplementary material for: Natural Killer Cell Activation Signature Identifies Cyclin B1/CDK1 as a Druggable Target to Overcome Natural Killer Cell Dysfunction and Tumor Invasiveness in Melanoma
Source: Pharmaceuticals (Basel). 2025 Apr 30;18(5):666. doi: 10.3390/ph18050666 (PMC12114673; doi:10.3390/ph18050666)
Supplement: Supplementary file 1 [file pharmaceuticals-18-00666-s001.zip › Supplemental Table 1.pdf]

Supplemental Table 1. Real-time qPCR primers used in this research.

Gene	Forward Primer	Reverse Primer
CCNB1	GACCTGTGTCAGGCTTTCTCTG	GGTATTTTGGTCTGACTGCTTGC
IL-6	AGACAGCCACTCACCTCTTCAG	TTCTGCCAGTGCCTCTTTGCTG
TGFB1	TACCTGAACCCGTGTTGCTCTC	GTTGCTGAGGTATCGCCAGGAA
CD274	TGCCGACTACAAGCGAATTACTG	CTGCTTGTCCAGATGACTTCGG
GAPDH	GTCTCCTCTGACTTCAACAGCG	ACCACCCTGTTGCTGTAGCCAA
