# Supplementary figures and images for: Natural Killer Cell Activation Signature Identifies Cyclin B1/CDK1 as a Druggable Target to Overcome Natural Killer Cell Dysfunction and Tumor Invasiveness in Melanoma
Source: Pharmaceuticals (Basel). 2025 Apr 30;18(5):666. doi: 10.3390/ph18050666 (PMC12114673; doi:10.3390/ph18050666)

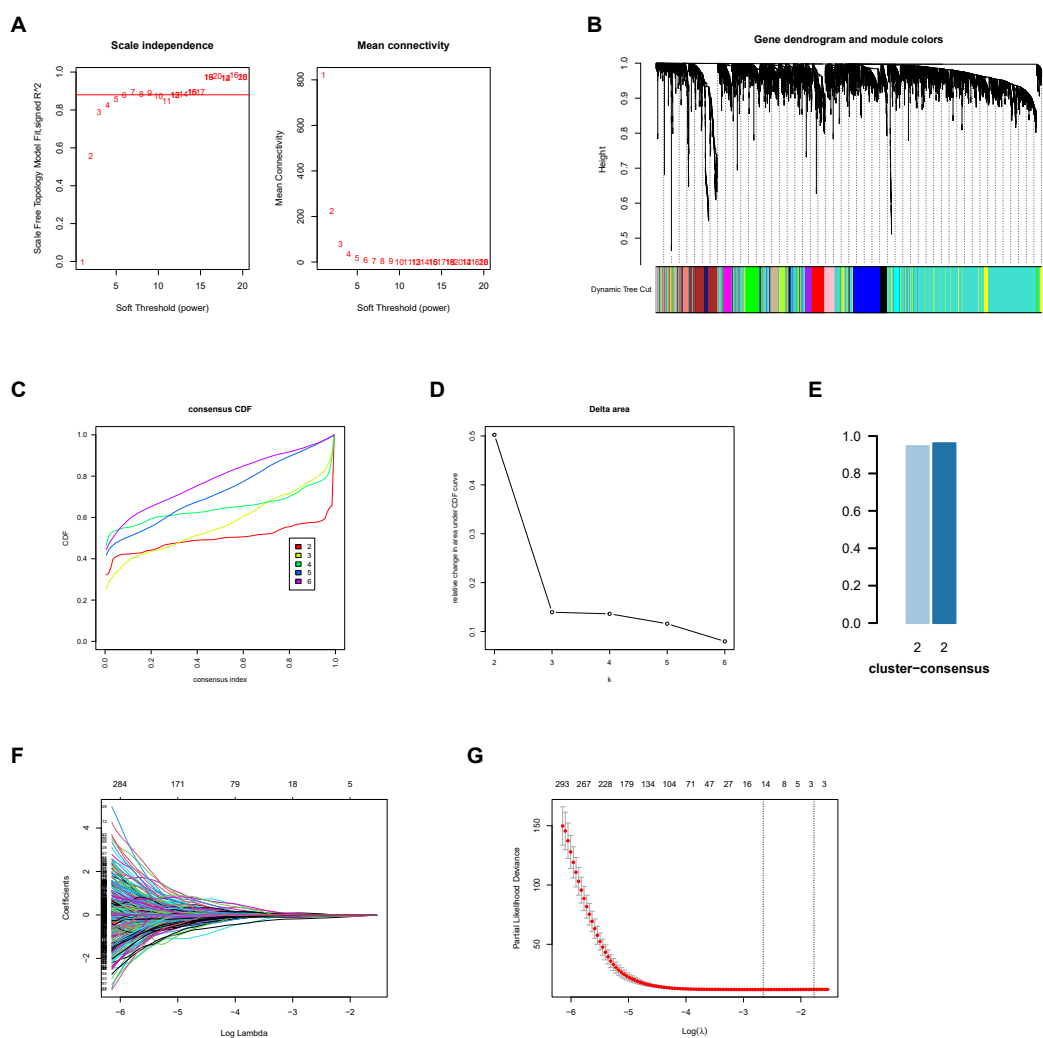

Supplement: Supplementary file 1 [file pharmaceuticals-18-00666-s001.zip › Supplemental Figure 1.pdf]
